# Inhibition of Exotoxin Production by Mobile Genetic Element SCC*mec*-Encoded *psm-mec* RNA Is Conserved in Staphylococcal Species

**DOI:** 10.1371/journal.pone.0100260

**Published:** 2014-06-13

**Authors:** Mariko Ikuo, Gentaro Nagano, Yuki Saito, Han Mao, Kazuhisa Sekimizu, Chikara Kaito

**Affiliations:** Laboratory of Microbiology, Graduate School of Pharmaceutical Sciences, The University of Tokyo, Hongo, Bunkyo-ku, Tokyo, Japan; University Hospital Münster, Germany

## Abstract

Staphylococcal species acquire antibiotic resistance by incorporating the mobile-genetic element SCC*mec*. We previously found that SCC*mec*-encoded *psm-mec* RNA suppresses exotoxin production as a regulatory RNA, and the *psm-mec* translation product increases biofilm formation in *Staphylococcus aureus*. Here, we examined whether the regulatory role of *psm-mec* on host bacterial virulence properties is conserved among other staphylococcal species, *S. epidermidis* and *S. haemolyticus*, both of which are important causes of nosocomial infections. In *S. epidermidis,* introduction of *psm-mec* decreased the production of cytolytic toxins called phenol-soluble modulins (PSMs) and increased biofilm formation. Introduction of *psm-mec* with a stop-codon mutation that did not express PSM-mec protein but did express *psm-mec* RNA also decreased PSM production, but did not increase biofilm formation. Thus, the *psm-mec* RNA inhibits PSM production, whereas the PSM-mec protein increases biofilm formation in *S. epidermidis.* In *S. haemolyticus,* introduction of *psm-mec* decreased PSM production, but did not affect biofilm formation. The mutated *psm-mec* with a stop-codon also caused the same effect. Thus, the *psm-mec* RNA also inhibits PSM production in *S. haemolyticus*. These findings suggest that the inhibitory role of *psm-mec* RNA on exotoxin production is conserved among staphylococcal species, although the stimulating effect of the *psm-mec* gene on biofilm formation is not conserved.

## Introduction

Pathogenic bacteria produce exotoxins that damage host immune cells to facilitate bacterial survival and proliferation in the host environment. Pathogenic bacteria also form a biofilm to resist host immune factors and antibiotics [1]. Understanding the molecular mechanisms of exotoxin production and biofilm formation is important for establishing therapeutic strategies against infectious bacterial diseases. Bacteria possess virulence factors encoded on the core genome and also acquire virulence factors by incorporating plasmids, phages, or transposons, which are known as mobile genetic elements [2]. Mobile genetic elements encode various virulence factors such as exotoxins and superantigens that directly interact with host factors [3], [4]. Mobile genetic elements also encode a regulatory factor against core genome encoded-virulence genes [5], [6], [7].

We recently found that the *psm-mec* gene locating in the mobile genetic element SCC*mec,* which carries antibiotic resistant genes, regulates the virulence properties of *S. aureus,* a serious human pathogen [8]. In the *S. aureus* core genome, the *agr* locus encodes *agrBDCA* and RNAIII, which regulate the expression of various virulence genes according to cell density [9]. The *agrA* gene encodes a positive transcription factor for phenol-soluble modulins (PSMs), which are cytolysins essential for *S. aureus* virulence [10], [11], [12]. The transcription product of *psm-mec* acts as a regulatory RNA to inhibit the translation of *agrA,* resulting in decreased PSMs production [13]. In contrast, the translation product of *psm-mec* stimulates biofilm formation [8]. The *psm-mec* gene exists in type-II and type-III SCC*mec* of hospital-associated methicillin-resistant *S. aureus* (HA-MRSA), but not in type-IV SCC*mec* of community-acquired MRSA with higher virulence than HA-MRSA [14], [15], [16]. Furthermore, 25% of HA-MRSA strains carry promoter-deficient *psm-mec* and produce higher amounts of a cytolytic exotoxin, PSMα3, than the strains carrying intact *psm-mec* [13]. These findings indicate that *psm-mec* is a genetic determinant of the virulence capacity of MRSA. The function of *psm-mec* to inhibit exotoxin production and to increase biofilm formation might contribute to alleviate excess damage to host animals and to survive in the host environment. The *psm-mec* exists not only in *S. aureus*, but also in other staphylococcal species [17]. It has remained unclear, however, whether the regulatory function of *psm-mec* is conserved among the staphylococcus species carrying SCC*mec,* which needs the conserved interaction between the mobile genetic element-encoded *psm-mec* and core genome encoded-virulence factors.

Most staphylococcal species other than *S. aureus* do not produce coagulase and are called coagulase-negative staphylococci. *S. epidermidis* is a commensal bacterium present on human skin surfaces that often contaminates catheters and other surgical implants [18], [19]. The recent emergence of methicillin- or vancomycin-resistant *S. epidermidis* is a serious clinical problem [20]. *S. haemolyticus* is also a commensal bacterium on human skin surfaces and in domestic animals [21] that causes various infectious diseases, including skin infections and meningitis in humans [21]. In particular, *S. haemolyticus* bacteremia in a neonatal intensive care unit and the emergence of drug-resistant *S. haemolyticus* were recent serious clinical issues [22], [23]. In the present study, we investigated the effect of *psm-mec* in *S. epidermidis* and *S. haemolyticus* and found that the inhibitory function of *psm-mec* RNA on exotoxin production was conserved among these species.

## Results and Discussion

### *psm-mec* Alters the Virulence Phenotype of *S. epidermidis*

**Figure 1:**
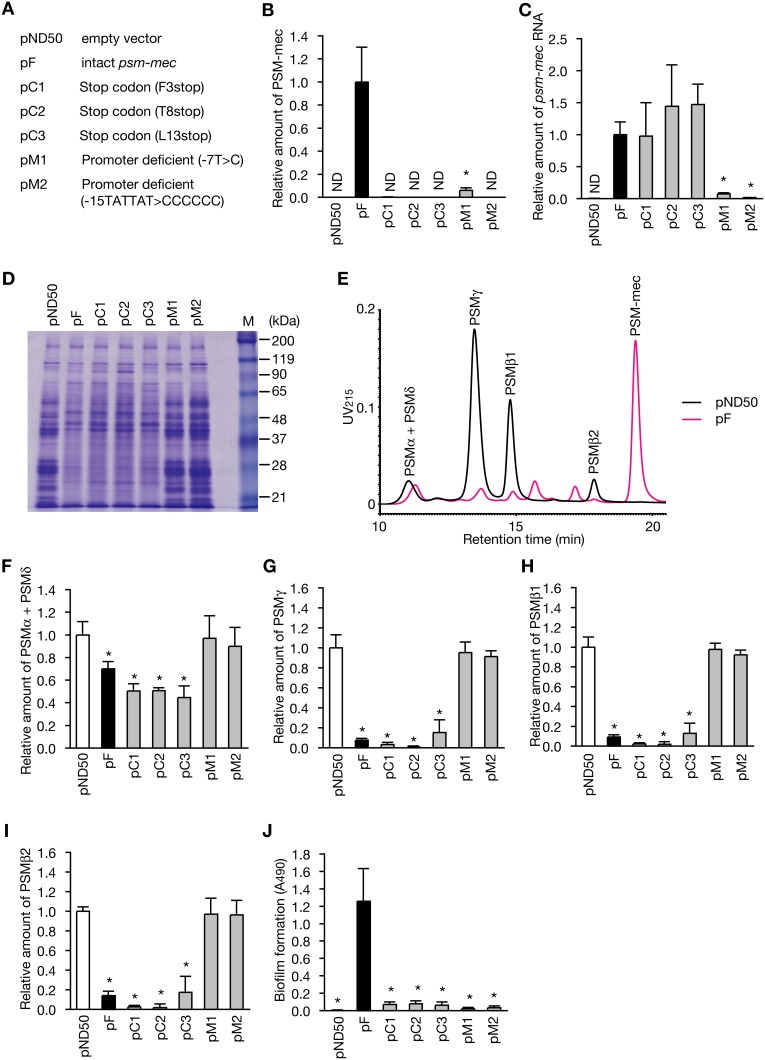
Alteration of virulence phenotype in the *psm-mec*-transformed *S. epidermidis*. (A) Schematic representation of the *psm-mec* mutations in pC1, pC2, pC3, pM1, and pM2 is shown. (B) The amount of PSM-mec protein was measured by reversed-phase HPLC in the *S. epidermidis* strains transformed with plasmids listed in (A). Data are means ± standard deviations from three independent experiments. Asterisks indicate Student’s t-test p value less than 0.05 between the pF-transformed stain and the others. ND means not detected. (C) The amount of *psm-mec* RNA was measured by quantitative RT-PCR in the *S. epidermidis* strains transformed with plasmids listed in (A). Data shown are means ± standard deviations from three independent experiments. Asterisk indicates Student’s t-test p value less than 0.05 between the pF-transformed stain and the others. ND means not detected. (D) Extracellular proteins of overnight culture of *S. epidermidis* ATCC12228 strain, which was transformed with pND50 as an empty vector, pF carrying intact *psm-mec*, or plasmids carrying mutated *psm-mec* (pC1, pC2, pC3, pM1, or pM2) was electrophoresed in a 10% SDS polyacrylamide gel and stained by Coomassie brilliant blue. A representative result from three independent experiments is shown. (E) Expression of PSMs in the *psm-mec*-transformed strain (pF, magenta line) or empty vector-transformed strain (pND50, black line) was analyzed by reversed-phase HPLC. The PSM species were identified by LC/ESI-MS. (F, G, H, I) The amounts of PSMα + PSMδ, PSMγ, PSMβ1, and PSMβ2 in the *S. epidermidis* strain that was transformed with pND50 as an empty vector, pF carrying intact *psm-mec*, or plasmids carrying mutated *psm-mec* (pC1, pC2, pC3, pM1, or pM2) were measured by reversed-phase HPLC. The vertical axis represents the relative value to the amount of PSMs in the pND50-transformed strain. Data are shown as means ± standard deviations from three independent experiments. Asterisks indicate Student’s t-test with a p value less than 0.05 between the pND50-transformed stain and others. (J) Biofilm formation of the *S. epidermidis* strains transformed with pND50 as an empty vector, pF carrying intact *psm-mec*, or plasmids carrying mutated *psm-mec* (pC1, pC2, pC3, pM1, or pM2) was measured. Data shown are means ± standard deviations from three independent experiments. Asterisks indicate Student’s t-test with a p value less than 0.05 between the pF-transformed stain and the others.

To examine whether the alteration of the *S. epidermidis* phenotype by *psm-mec* is caused by the transcription product or the translation product of *psm-mec*, we transformed *S. epidermidis* with *psm-mec* carrying either a stop codon mutation or a deficient promoter, which we previously used in *S. aureus* [8] (Fig. 1A). The strains transformed with pC1, pC2, or pC3, which carries a mutated *psm-mec* with a stop codon, did not produce PSM-mec protein (Fig. 1B), but expressed an amount of *psm-mec* RNA indistinguishable from that of the intact *psm-mec*-transformed strain (Fig. 1C). In contrast, the strains transformed with pM1 or pM2, which carried the promoter-deficient *psm-mec*, expressed little amount of *psm-mec* RNA or PSM-mec protein (Fig. 1B and 1C). These findings suggest that the translation of *psm-mec* was inhibited by pC1, pC2, and pC3, whereas the transcription of *psm-mec* was inhibited by pM1 and pM2 in *S. epidermidis*.

We transformed *S. epidermidis* ATCC12228 strain, which does not possess *psm-mec,* with a plasmid carrying *psm-mec* (pF). Analysis of extracellular proteins by sodium dodecyl sulfate (SDS)-polyacrylamide gel electrophoresis revealed that the expression pattern of extracellular proteins was altered in the *psm-mec*-transformed strain compared with the vector (pND50)-transformed strain (Fig. 1D). *S. epidermidis* secretes PSMs that are involved in biofilm maturation and have cytolytic activity against mammalian cells [24], [25]. High-performance liquid chromatography (HPLC) analysis revealed that the introduction of *psm-mec* decreased the amount of PSMs in culture supernatants (Fig. 1E). In the *psm-mec-*transformed strain, the combined amount of PSMα and PSMδ, which were not separated by our HPLC conditions, was decreased to 70% of that in the vector-transformed strain (Fig. 1F). The amounts of PSMγ, PSMβ1, and PSMβ2 in the *psm-mec-*transformed strain were decreased to 10% of that in the vector-transformed strain (Fig. 1G, 1H, and 1I). In contrast, the *psm-mec-*transformed strain exhibited increased biofilm formation compared with the vector-transformed strain (Fig. 1J). These findings suggest that *psm-mec* alters the expression pattern of extracellular proteins, decreases the expression of PSMs, and stimulates biofilm formation in *S. epidermidis.*

The mutated *psm-mec* with a stop codon in pC1, pC2, and pC3 retained the activities to alter the expression pattern of extracellular proteins and to decrease the amount of PSMs (Fig. 1D, 1F, 1G, 1H, and 1I). In contrast, the mutated *psm-mec* with a stop codon lost the ability to increase biofilm formation (Fig. 1J). The promoter-deficient *psm-mec* in pM1 and pM2, which did not express either *psm-mec* RNA and PSM-mec protein, did not induce the phenotypic alteration of *S. epidermidis,* which was caused by intact *psm-mec* (Fig. 1D, 1F, 1G, 1H, 1I, and 1J). These results suggest that the *psm-mec* translation product promotes biofilm formation, whereas the *psm-mec* transcription product inhibits PSM expression and alters the expression pattern of extracellular proteins in *S. epidermidis*.

### *psm-mec* Alters the Virulence Phenotype of *S. haemolyticus*

**Figure 2:**
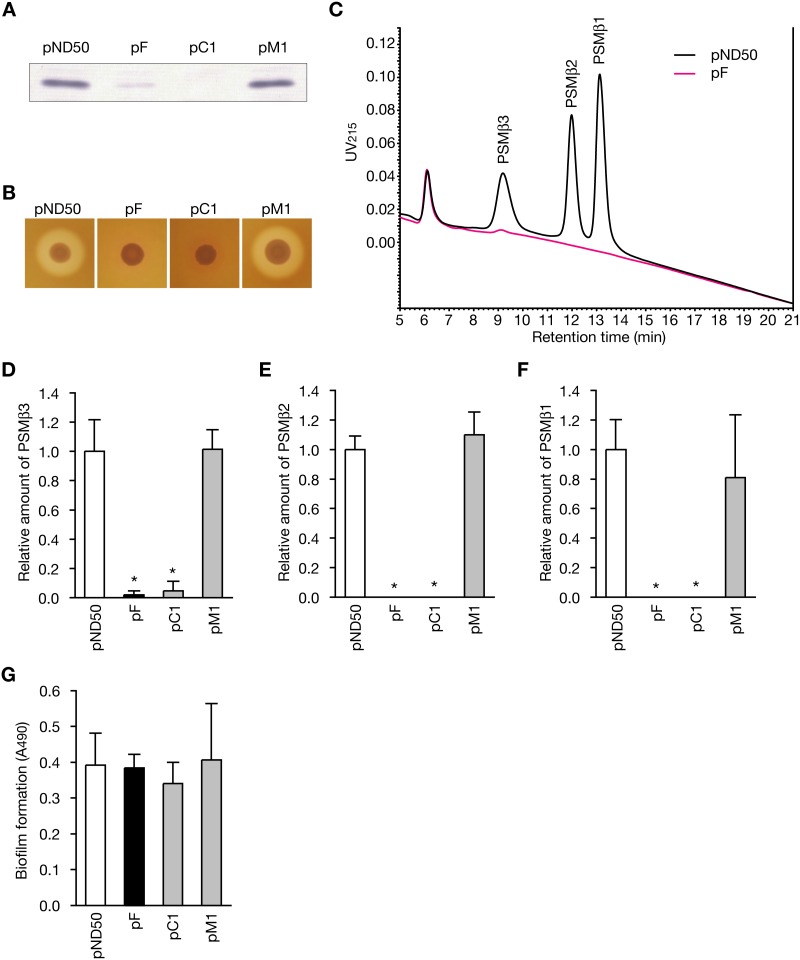
Alteration of virulence phenotype of the *psm-mec*-transformed *S. haemolyticus*. (A) AgrA expression in *S. haemolyticus* strain transformed with pND50 as an empty vector, pF carrying intact *psm-mec*, pC1 carrying a stop codon-mutated *psm-mec*, or pM1 carrying a promoter-deficient *psm-mec* was examined. Protein (2.8 µg) was electrophoresed in each lane and subjected to Western blotting using anti-AgrA IgG. A representative result from two independent experiments is shown. (B) Hemolysin production of *S. haemolyticus* strain transformed with pND50, pF, pC1, pr pM1 was measured on tryptic soy agar plates containing 5% sheep erythrocytes. A color-changed region around the colonies reflects the lysis of erythrocytes. (C) PSMs in *S. haemolyticus* strains transformed with pND50 or pF were detected by reversed-phase HPLC. Respective PSM species were identified by LC/ESI-MS. (D, E, F) The amounts of PSMβ3, PSMβ2, and PSMβ1 in the *S. epidermidis* strain transformed with pND50, pF, pC1, or pM1 were measured by reversed-phase HPLC. The vertical axis represents the relative value to the amount of PSMs in the pND50-transformed strain. Data shown are means ± standard deviations from three independent experiments. Asterisks indicate a Student’s t-test p value less than 0.05 between the pND50-transformed stain and the others. (G) Biofilm formation of the *S. haemolyticus* strain that was transformed with pND50, pF, pC1, or pM1 was examined. Data shown are means ± standard deviations from three independent experiments.

The *agr* locus encoding *agrBDCA* and RNAIII is involved in regulating *S. aureus* virulence properties [9]. Although the *agr* function is not elucidated in *S. haemolyticus*, the sequence of *agrBDCA* is conserved in *S. haemolyticus* [26]. We examined whether *psm-mec* decreases the expression of *agrA* in *S. haemolyticus*. Introduction of *psm-mec* decreased the amount of AgrA in the *S. haemolyticus* JCM2416 strain that does not carry *psm-mec* (Fig. 2A). In addition, the *psm-mec*-transformed strain decreased hemolysin production (Fig. 2B). Although there are no reported analyses of the PSMs of *S. haemolyticus,* genome analysis revealed the presence of genes encoding PSMβ1, PSMβ2, and PSMβ3 [27]. We performed HPLC analysis of the culture supernatant of *S. haemolyticus* and identified several molecules with absorbance at 215 nm within the retention time in which the PSMs of *S. aureus* and *S. epidermidis* are eluted (Fig. 2C). The molecules at 9, 12, and 13 min were identified as PSMβ3, PSMβ2, and PSMβ1 by liquid chromatography/mass spectroscopy analysis,respectively (Fig. 2C). The *psm-mec*-transformed strain exhibited decreased amounts of PSMβ3, PSMβ2, and PSMβ1 (Fig. 2C, 2D, 2E, and 2F). Introduction of the mutated *psm-mec* with a stop codon (pC1) also decreased the amounts of AgrA, hemolysin, and PSMs (Fig. 2A, 2B, 2D, 2E, and 2F). In contrast, introduction of the promoter-deficient *psm-mec* (pM1) did not cause the phenotypic alterations (Fig. 2A, 2B, 2D, 2E, and 2F). Thus, the *psm-mec* transcription product decreased the expression of AgrA, hemolysin, and PSMs in *S. haemolyticus.*

The *psm-mec*-transformed strain of *S. haemolyticus* exhibited the same level of biofilm formation as the vector-transformed strain (Fig. 2G). Introduction of either the mutated *psm-mec* with a stop codon (pC1) or the promoter-deficient *psm-mec* (pM1) did not alter the biofilm formation by *S. haemolyticus* (Fig. 2G). These findings suggest that *psm-mec* does not affect the biofilm formation by *S. haemolyticus.*

### Conclusions

In this study, we revealed that the inhibitory effect of the *psm-mec* transcript against exotoxin production is conserved in *S. epidermidis* and *S. haemolyticus.* This is the first report to reveal that the regulatory role of a mobile genetic element-encoded factor on host bacterial virulence is conserved among the species incorporating the mobile genetic element. Because the SCC*mec* carrying *psm-mec* is widely observed among staphylococcal species, including *S. aureus, S. epidermidis, S. haemolyticus, S. hominis, S. pseudintermedius, S. saprophyticus, S. fleurettii, S. vitulinus,* and *S. simulans* [14], [17], the inhibitory effect of *psm-mec* against exotoxin production is assumed to confer conserved advantages to staphylococcal bacteria in their survival in host animals and may cause a positive effect on the dissemination and maintenance of SCC*mec*.

In *S. haemolyticus,* the intact *psm-mec* or the stop codon-mutated *psm-mec* decreased the amount of AgrA. Thus, *psm-mec* RNA is assumed to inhibit translation of AgrA, as in the case of *S. aureus* [13]. We were not able to examine the effect of *psm-mec* on AgrA expression in *S. epidermidis* ATCC12228 because the expression level was too low. Based on the reports that the *agr* locus is required for PSM expression in *S. epidermidis* [28], [29] and our result that *psm-mec* RNA inhibited PSM expression of *S. epidermidis*, we assume that *psm-mec* RNA inhibits AgrA expression in *S. epidermidis.* Because the *agr* locus is conserved among staphylococcal species [26], the inhibition of *agr* function by *psm-mec* RNA is probably conserved among staphylococcal bacteria carrying SCC*mec* containing *psm-mec*. Exotoxin expression promoted by the *agr* system is energy consuming, resulting in spontaneous mutants of the *agr* locus preferentially growing in environments that do not require exotoxin expression [30]. For example, in *S. aureus* under laboratory conditions, spontaneous mutations frequently occur in the *agr* locus [30], [31], [32]. In addition, many *agr*-mutated strains of *S. aureus* have been isolated from clinical samples [33], [34], [35]. The *agr-*null mutant of *S. aureus* has high fitness in the presence of sublethal amounts of antibiotics [36]. The inhibition of the *agr* system by *psm-mec* might contribute to less energy-consuming and fitness to the antibiotic environment, in addition to avoiding too much damage to the host.

Introduction of *psm-mec* promoted biofilm formation by *S. epidermidis,* whereas it did not alter biofilm formation by *S. haemolyticus*. Thus, the effect of *psm-mec* on biofilm formation was not conserved among staphylococcal species. Based on the results that the stop codon-mutated *psm-mec* lost the ability to promote biofilm formation in *S. epidermidis*, PSM-mec protein was responsible for promoting biofilm formation of *S. epidermidis*. In *S. haemolyticus,* PSM-mec protein, which should elute at around 19 min in the HPLC analysis, was not detected in the *psm-mec*-transformed strain (Fig. 2C). The lack of detectable PSM-mec protein is a possible reason for the non-promoting effect of the *psm-mec* gene on *S. haemolyticus* biofilm formation. Further studies are needed to clarify the molecular mechanism of PSM-mec expression in *S. haemolyticus* and its conservation among other staphylococcal species.

## Materials and Methods

### Bacterial Strains, Plasmids, and Culture Conditions

*S. aureus* RN4220 strain was used as a host for pND50 and its derivatives. Plasmids were extracted from the RN4220 strain and used for the transformation of *S. epidermidis* ATCC12228 [37] or *S. haemolyticus* JCM2416 [38]. These strains were transformed by electroporation as in the case of *S. aureus* [39]. *S. epidermidis* and *S. haemolyticus* strains were aerobically cultured in tryptic soy broth at 37°C. When culturing the transformed strains, chloramphenicol was added to the broth to maintain plasmids.

### Evaluation of Biofilm Formation

*S. epidermidis* and *S. haemolyticus* strains were cultured in tryptic soy broth containing 0.25% glucose in 96-well polystyrene microplates (Cat No. 3860-096, Iwaki, Tokyo, Japan) at 37°C for 3 days. After removing the cultures, the plate was stained with 0.1% safranin. Absorbance at 490 nm was measured using a microplate reader (MTP300, CORONA, Ibaraki, Japan).

### Measurement of the Amount of PSMs

Fifty-microliters of overnight cultures of *S. epidermidis* or *S. haemolyticus* strains were inoculated into 5 ml of tryptic soy broth and aerobically cultured at 37°C for 18 h or 14 h. The culture supernatants were dried using a centrifugal evaporator. The precipitates were dissolved in 40% acetonitrile and the soluble fraction was dried. The precipitates were dissolved in water and analyzed in reversed phase HPLC using SOURCE 5RPC ST 4.6/150 column (GE Healthcare, Tokyo, Japan) and 50% acetonitrile in 0.1% trifluoroacetic acid for 3 min and a water/acetonitrile gradient in 0.1% trifluoroacetic acid from 50% to 90% acetonitrile for 20 min at a flow rate of 1 ml/min (600E, Waters, Milford, MA). Absorbance at 215 nm was measured using a 2998 Photodiode Array Detector (Waters). Each PSM species was identified by liquid chromatography/electrospray-ionization mass spectroscopy (LC 1100 series, Agilent Technologies, Santa Clara, CA; ESI-MS, Bio-TOFQ, Bruker Daltonics, Billerica, MA) and the predicted molecular masses of PSMs [25], [27]. *S. epidermidis* PSMα and PSMδ were eluted at the same retention time in this assay condition.

### Measurement of the Amount of *psm-mec* RNA

*S. epidermidis* strains were aerobically cultured to A_600_ = 1 at 37°C and the cells were collected by centrifugation at 20,000 *g* for 1 min. The cells were treated with RNAprotect Bacteria Reagent (Qiagen) and the total RNA was extracted using an RNeasy Mini Kit (Qiagen). RNA was reverse-transcribed to cDNA using Multiscribe Reverse Transcriptase (Roche). Quantitative polymerase chain reaction (PCR) was performed using the cDNA, SYBR Premix ExTaq (Takara Bio, Tokyo, Japan), and primers for 16 S rRNA or *psm-mec* according to the previously described method [13]. Signals were detected using the Step One Plus Real Time PCR System (Applied Biosystems, Tokyo, Japan).

### Detection of *S. haemolyticus* AgrA

*S. haemolyticus* AgrA was detected according to the previously described method [13]. Briefly, *S. haemolyticus* was aerobically cultured for 15 h at 37°C and the cells were collected by centrifugation at 20,000 *g* for 1 min. The cells were lysed in lysis buffer (10 mM Tris-HCl [pH 8.0], 1 mM EDTA, 10 µg/ml lysostaphin) at 37°C for 30 min. The samples were sonicated and centrifuged at 20,000 *g* for 5 min. The protein concentration of the supernatants was determined by the Bradford assay. The protein was electrophoresed in a 15% SDS polyacrylamide gel and blotted onto a polyvinylidene difluoride membrane (Immobilon-P, Millipore). The membrane was subjected to detection of AgrA by using anti-AgrA antibody.
